# Predictive genetic testing for Huntington's disease: Exploring participant experiences of uncertainty and ambivalence between clinic appointments

**DOI:** 10.1002/jgc4.1911

**Published:** 2024-05-13

**Authors:** L. M. Ballard, S. Doheny, R. Dimond, A. M. Lucassen, A. J. Clarke

**Affiliations:** ^1^ Clinical Ethics, Law & Society (CELS), Primary Care, Population Sciences and Medical Education Aldermoor Health Centre, Aldermoor Close Southampton UK; ^2^ Institute of Medical Genetics, Division of Cancer & Genetics School of Medicine, Cardiff University Cardiff UK; ^3^ School of Social Science Cardiff University Cardiff Wales UK; ^4^ Wellcome Trust Centre for Human Genetics University of Oxford Oxford UK

**Keywords:** ambivalence, decision making, Huntington's disease, predictive genetic testing, uncertainty

## Abstract

Ambivalence and uncertainty are key themes throughout the psychology of healthcare literature. This is especially so for individuals at risk of Huntington's disease (HD) deliberating the decision to undergo genetic testing because there is currently no treatment that modifies disease progression. A better understanding of the experience of making a decision about genetic prediction will help practitioners support and guide individuals through this process. Our aim was to capture participants' experiences of uncertainty and ambivalence in between their genetic counseling appointments. We explored these issues through the experiences of nine participants who were referred for predictive HD testing at four regional genetics services in England and Wales. Data consisted of recordings of clinic consultations, diaries, and an in‐depth interview conducted at the end of the testing process. Data were analyzed thematically. Four themes were identified representing four possible futures, each future dependent on the decision to undergo testing and the result of that test. Our results showed that participants, as well as attending more to a future that represents their current situation of not having undergone predictive testing, also attended more to a distant future where a positive predictive result is received and symptoms have started. Participants attended less to the two futures that were more immediate once testing was undertaken (a future where a positive result is received and symptoms have not started and a future where a negative result is received). The use of diaries gave us a unique insight into these participants' experiences of ambivalence and uncertainty, psychological distress, and the emotional burden experienced. These findings help inform discussions within the clinic appointment as well as encourage researchers to consider diary use as a method of exploring what happens for individuals outside of clinical encounters.

**What is known about this topic**

Uncertainty and ambivalence are common and often distressing experiences for individuals who are undergoing genetic testing. This is especially the case in predictive testing for HD due to there being no treatment that modifies disease progression.

**What this paper adds to the topic**

Our findings cast light on how participants focused on the most distant of the possible futures resulting from a predictive test for HD. We also successfully utilized the novel diary elicitation method as a way of accessing participant experiences beyond the brief clinical encounters that punctuated the testing journey.

## 1 INTRODUCTION

Managing uncertainty is a key theme in the psychology of healthcare literature (Sweeny & Cavanaugh, 2012)—especially in relation to genetic testing (Rew et al., 2010). Uncertainty experienced in relation to genetic tests is particularly prominent and is generated by multiple sources (Newson et al., 2016). For example, Newson et al., 2016 categorize this uncertainty into probability (when future outcomes are characterized by an inherent unpredictability); ambiguity (when information or evidence is vague, opinions conflict, or when there is a lack of knowledge); and complexity (when the available information contains complexities or ambiguities that hinder clear understanding). In the case of a presymptomatic genetic test for Huntington's disease (HD), a positive result usually indicates certainty given the age‐related but high penetrance. Within that certainty, however, lies some uncertainty relating to when symptoms will develop and the severity of those symptoms (Evans et al., 2001). As the above highlights, the experience of uncertainty is not reserved for the small amount of time individuals are in contact with the healthcare environment. Methodologically, this poses a challenge; how can individuals' experiences of uncertainty be explored and learned from to better support them within the health system, when most of these experiences occur beyond the clinic?

To explore individual experiences of uncertainty and ambivalence, we chose to focus on HD because, while there are therapeutic treatments to improve symptoms, there is currently no cure. This means that genetic health professionals in the United Kingdom offer a nondirective approach: individuals are helped to make the decision that is best for them rather than any particular predetermined choice (Elwyn et al., 2000).

**FIGURE 1:**
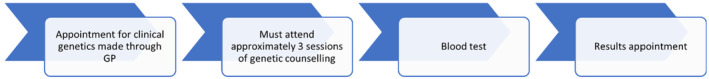
Genetic counseling pathway for HD (UK; Huntington's Disease Association (HDA), 2020).

HD is characterized by progressive motor, cognitive, and psychiatric impairments (Novak & Tabrizi, 2010). Involuntary and irregular movements and loss of balance are common early symptoms. Each offspring of an affected person has a 50:50 chance of inheriting the genetic variant. Individuals can access predictive testing through clinical genetics services, usually from the age of 18 as per current guidelines (Macleod et al., 2013). HD develops as a result of an expansion in the number of CAG triplet repeats within the *HTT* gene (Novak & Tabrizi, 2010). Individuals with 40 or more repeats will develop HD; there is somewhat reduced penetrance for individuals with 36–39 repeats. The mean age of onset is ~40 years. A referral to consider predictive testing in a family with HD may be triggered by factors including the belief that symptoms have started, wanting to start a family, or the wish to resolve uncertainty (Smedley & Coulson, 2019; Tillerås et al., 2020) with individuals deliberating the decision whether to undergo predictive genetic testing. The lack of treatment and the eventually fatal nature of the disease mean that there has been reluctance to provide predictive testing for HD without preparatory counseling, with the aim of confirming that a person is “ready” to receive their result or helping them to become so (Macleod et al., 2013; see Figure 1 for a typical genetic counseling process for HD in the United Kingdom).

It could be said that all “healthy” people inhabit a space between health and disease. Inhabiting the zone of risk between health and disease has been conceptualized as existing in an in‐between state, a liminal or ambiguous space between health and illness (Kavanagh & Broom, 1998). Liminality—the term “limen” in Latin means threshold—describes the psychological process of moving from one space to the threshold of another (Larson, 2014), it is “a state of ‘no longer/not yet’” where previous narratives no longer apply and the new narrative has not quite arrived (Sutton, 2017); it is about “life in between” (Koutri & Avdi, 2016, p. 80). For individuals with a family history of HD, the risk is not vague and remote but has been made more concrete and brought closer through awareness of their family history and/or predictive genetic testing (Cox & McKellin, 1999). Furthermore, the risk may not be perceived by individuals, clinicians, and laboratory scientists in the same way. For example, while Mendelian models offer objective odds, lay understandings consider social factors and familial interactions, shaping risk in a fluid, creative, and subjective manner. Family dynamics, geographic proximity, social ties, the complexity of everyday experiences, and the ability to reflect on uncertainties define lay risk perspectives (Cox & McKellin, 1999).

Ambivalence plays a part here too. Defined as “the simultaneous existence of contradictory feelings and attitudes” (American Psychological Association, 2023), ambivalence is often psychologically unpleasant, leading to a desire toward resolution through making a decision or taking action (Reich & Wheeler, 2016). Individuals deliberating the decision to undergo predictive genetic testing suggest that ambivalence is worse than knowledge either way (Lewit‐Mendes et al., 2018; Smedley & Coulson, 2019). However, the individual may be anxious in case this claim is interpreted as performative (a mere tactic to access testing). The counselor wants to be confident the individual believes that a positive test result would be better than continued uncertainty (Sarangi et al., 2005). Preparatory counseling therefore functions to resolve ambivalence and uncertainty, with the irreversible nature of taking the test and finding out the results contributing to the ambivalence it generates.

The decision to undergo predictive testing for HD can impact negatively on familial relationships when relatives are unsupportive of the decision, not had testing themselves, or are in denial about their own risks (Stuttgen et al., 2020). For individuals, and their families, finding out that they have inherited the genetic variant can be devastating (Novak & Tabrizi, 2010), therefore it is easy to imagine why the decision about testing can be incredibly difficult. Some individuals undergoing the testing process report experiencing and struggling with anxiety, depression, fear, hopelessness, isolation, and loneliness, all of which encroach on their daily activities and affect their plans for the future (Smedley & Coulson, 2019; Tillerås et al., 2020). Even if the decision to test is straightforward, uncertainty and a focus on negative outcomes when it comes to receiving and sharing medical test results can also trigger unpleasant thoughts and feelings (Cox & McKellin, 1999; Sweeny & Cavanaugh, 2012).

The decision to undergo predictive testing for HD is a complex and much studied one, but with few examples of prospective exploration of the process (Cox & McKellin, 1999; Ibisler et al., 2017; Keenan et al., 2015; Sarangi et al., 2005) and none have gathered diary‐based data. Our aim was to capture participants' experiences between healthcare interactions, with a focus on accounts of uncertainty and ambivalence.

## 2 METHOD

### 2.1 Participants

In this paper, we report findings from nine participants who completed and returned a diary, had at least one clinic appointment recorded, and participated in a final interview. These nine were recruited from 17 individuals from HD families referred to the study via health professionals from four regional genetics centers in England and Wales. All participants in this sample were at 50% risk. Participants ranged between 16 and 50 years of age (see Table 1 for participant details). Although it is recommended that the minimum age of predictive genetic testing for HD is 18 years (Macleod et al., 2013), one participant was under 18 years. The nine participants had varying experiences of HD in their families, with many becoming aware of their risk of HD after a parent was diagnosed following the development of clinical symptoms. Ethical approval for human subject research was gained through a Local Research Ethics Committee, the participating NHS organizations in England and Wales, and the Health Research Authority. No rewards were offered for participation.

**TABLE 1:** Participant identifier and demographics (all participants in this sample underwent predictive genetic testing for HD).

Pseudonym	Kayleigh	Aiden	Jacob	Leah	Rosie	Tom	Sally	Aaron	Dee
Gender	F	M	M	F	F	M	F	M	F
Age range	16–20	21–30	21–30	21–30	21–30	21–30	31–40	41–50	41–50

### 2.2 Procedure

**FIGURE 2:**
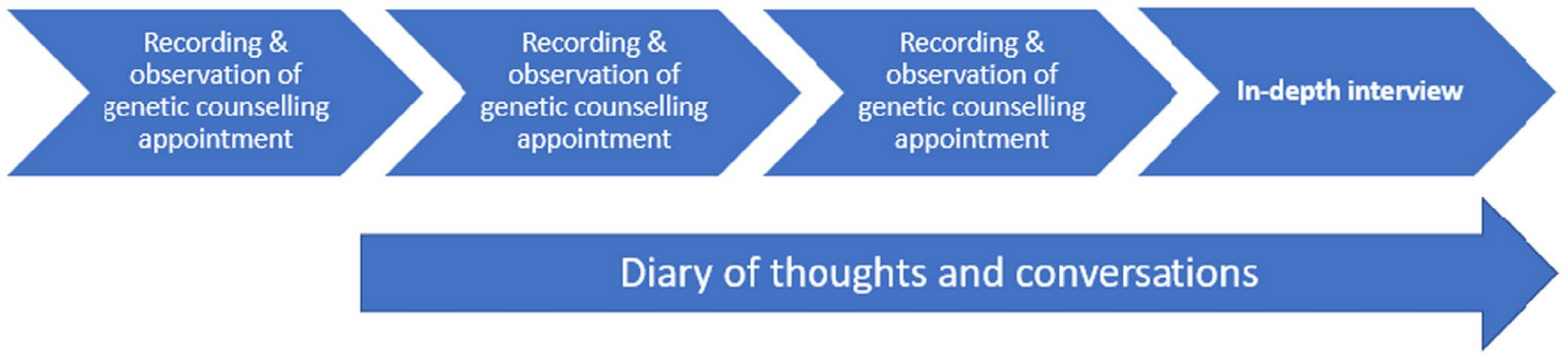
Data generation.

Once referred to the study, individuals were sent written information about the research by the clinical admin team along with their appointment letter. On arrival in clinic, individuals were asked by the clinical team whether they had received the information and whether they were willing to meet the researcher who could further explain the study. Oral consent was then sought, and their appointment was audio recorded. Written consent to continue in the project, and for the researchers to keep and use the recording, was sought after this initial appointment. This consent process was agreed upon with the Health Research Authority after some months of using a more complex consent process. It was deemed important that an understanding of the process of genetic counseling obtained in the first appointment would help the participant in their decision whether to proceed with the research element. Any subsequent clinical appointments were audio recorded, including result appointments if deemed appropriate by the individual and the clinician (see Figure 2 for the data generation phases).

After written consent was received, participants were asked if they would keep a reflective diary of their thoughts and conversations regarding their deliberation and experiences. Researcher‐directed diaries in relation to health and illness have often been overlooked as a research method (Milligan & Bartlett, 2019), but have long been appreciated as offering access to harder‐to‐reach areas of people's lives (Elliott, 1997; Hilário & Augusto, 2023). Sometimes called “solicited,” researcher‐directed diaries involve participants recording their thoughts, feelings, and experiences regarding a topic predetermined by the researcher (Braun & Clarke, 2013). Participants were offered a paper diary or Dictaphone to record their diary entries but were also encouraged to choose any medium they felt comfortable with (email, text, and mobile phone voice recording). Diaries were recorded in a number of ways including handwritten diaries, text messages, and audio diaries. One participant was already keeping a diary and transcribed from that into a diary kept specifically for this study. Participants were not given specific guidance about what to record or when to complete an entry, but they were provided with a “Technical Information” sheet which included information about different ways to record thoughts and some ideas about what to record.

Some participants returned their diaries at the final clinic appointment, others by post (the study team sent a stamped, self‐addressed envelope) or in person at the closing interview. Once we received a physical diary, the team made a photocopy of the diary and returned the original to the individual by post. Those who kept their diaries electronically, forwarded these by email or text message. If diary entries raised concerns or distress, this was discussed at the earliest opportunity with members of the study team and/or wider clinical team. Once the participant had received their genetic test results, or the participant decided to not undergo testing, participants were invited for an in‐depth interview. Interviews were semi‐structured using a topic guide, consisting of standard questions asked of all participants as well as tailored questions based on diary entry and clinic consultation data. The interview data have formed the focus of a separate article (Dimond et al., 2022).

### 2.3 Analysis

**FIGURE 3:**
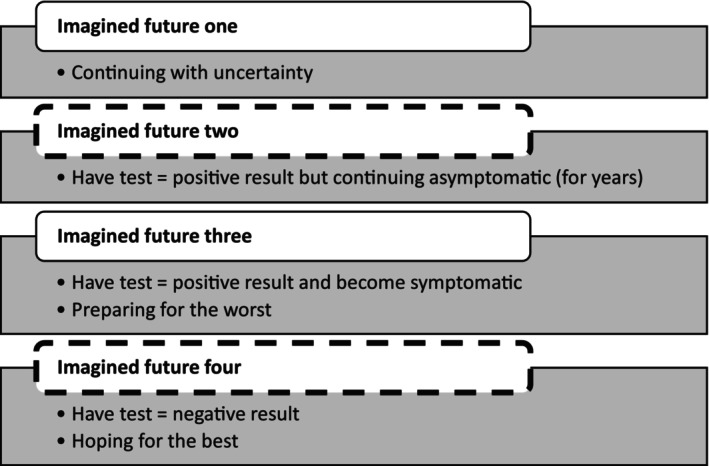
Thematic map (‐ ‐ ‐ indicates themes that were less present in the data).

Transcribed clinic consultations, diaries, and interviews, along with typed‐up field notes, were analyzed inductively using thematic analysis (TA) based on the Braun and Clarke (2006) method, and all themes were identified through reading and re‐reading of the texts. Through the inductive analysis, we identified the overarching themes of the multiple futures. There was a deductive element to the analysis, as previous literature and experience informed the exploration of ambivalence and uncertainty as concepts within the analysis. Using NVivo 12, transcripts were coded based on the initial themes. Each code was systematically reviewed to ensure quotes were consistent. Further analysis was conducted on each theme to identify overarching themes, connections, and missing themes (see Figure 3). LMB had experience of using TA, and analysis was discussed with members of the research team, which included clinicians and social scientists, to discuss meaning, interpretation, and categorization. As part of the process of analytical thinking, we considered what was not as present in the data, in recognition of “what is not in the data can be as important as what is present” (Ewart & Ames, 2020 p. 63). Therefore, Figure 3 also contains themes that are logically related to others but have less data to support them.

## 3 RESULTS

The analysis resulted in four themes which focused on imagining a particular future. The first represented continuing with uncertainty, reflecting the individual's current situation of not having undergone testing and their decision not to be tested (yet). The second was an imagined future where they had the test, received a positive (i.e., adverse, “bad news”) result, and had not yet started to experience symptoms of HD. The third was an imagined future where they had the test and received a positive result but were experiencing symptoms of HD. The fourth was that of undergoing testing and receiving a negative (favorable, “good news”) result and includes data on “hoping for the best.” Each quote has the data source indicated at the end in brackets along with the participant's pseudonym.

### 3.1 Imagined future 1: Continuing with uncertainty

Participants imagined future 1 as a continuation of their current reality, not as one they were contemplating but rather as an imaginary future that helped them deliberate the decision to have the test. Future 1 was a present and future marked by uncertainty; a present they knew only too well.

[E]very time you drop something, forget something, trip over, do something. [I'm worrying] ‘am I getting old, is there something else wrong with me, or have I started getting HD?’ (Dee, Clinic)

Future 1 describes how participants were coming upon the limits of their capacities to live with the uncertainties and ambivalence, which was also illustrated by their decision to initiate the testing process. It appeared that continuing into the future in the way they were managing at present was no longer tenable. Some may have felt ambivalent toward having a predictive genetic test for some time and may indeed still be feeling that way. Aiden described having a previous clinic appointment where he decided he was not ready to go through with testing. Prompted by his dad's decline in health, he described how it had been on his mind resulting in revisiting the testing process.

Just the way that I've seen my dad recently, over the past six months he's gone downhill, and it's playing on my mind, I would rather find out now rather than later. (Aiden, Clinic)

As Aiden described, participants appeared to feel that continuing uncertainty would be more stressful than knowing their result—be that positive or negative—and would not allow them to plan or prepare. And Kayleigh expressed her situation of uncertainty as potentially “more stressful than the actual end result.” Some participants appeared to have not experienced uncertainty until a life event prompted reflection, as was the case for Aiden who had witnessed his father's decline. For Tom, the prompt was the need to plan for the future. Once armed with “the facts,” Tom said in his clinic appointment that “I'd rather have the certainty of it, and then I could plan my life and move on, I don't want to always have this cloud lingering over me.” Ambivalence and uncertainty appear to be inextricably linked for these participants and the ambivalence experienced is more about the close link among imagining, predicting, and preparing for future certainty.

Aaron, in his diary entry, offered an insight into his ambivalence about having the test. In the quote below, he appears to be considering the future he thinks will happen rather than deciding whether to proceed with testing for HD when he says, “I'm completely ambivalent in this.” He described an unpleasant feeling when holding the futures in his mind simultaneously as the 50:50 chance of either result made it impossible to make any distinction between the likelihood of each future becoming a reality. Imagining both futures appeared mentally effortful and exhausting, which in itself might intensify the ambivalence.

When I do start thinking about it, it's quite unpleasant. Because I still can't fathom the idea of it being 50:50. And the two scenarios going through my head about being given the result are superimposed on each other. I've had a parallel universe version of myself experience one side of it, then the other side. I'm completely ambivalent in this. Very unpleasant. It's probably one of the crappiest odds you could ever come across. (Aaron, Diary)

Sally recorded in her diary that she was not even contemplating uncertainty, and leaving it to a higher power: “instead of feeling uncertain, I am leaving it with my God to guide me through my tests.” This reference to a spiritual frame is significant as participants in this sample did not mention this in their clinic consultations.

Living within a family with HD means that many participants have seen how others deal with ambivalence and uncertainty. Leah stated in her diary that her partner and parents had “convinced” her that living with the uncertainty was preferable and she theorized that it may be because of their own ways of coping. To gain some certainty and control she resorted to planning her end of life. This led Leah to conclude that knowing would be the “healthier” option for her.

Probably because they couldn't cope/didn't think it mattered yet, [partner] and my parents convinced me to live in ‘responsible ignorance’ and find out in a few years when I want children. The problem was, in the meantime I convinced myself I had it and was going to die at 40 as a coping mechanism and had planned out my suicide – it was all so that I felt some sense of control over my life. I think this was unhealthier than just knowing. (Leah, Diary)

In this theme, the diary data add context and emotional richness to the data from clinic consultations. We become privy to the contrasting views of a relative that spurred a participant toward knowing, spiritual frames that ease the cognitive load of making the “right” decision, and the vivid discomfort of imagining multiple futures. These diary data “bring to life” the effort and emotional labor participants are putting into, and coming to terms with, their decision.

Whereas imagined future 1 focuses on deliberating the decision to have the test, futures 2, 3, and 4 relate more to exploring the test outcome.

### 3.2 Imagined future 2: Take a test, receive a positive result, and remain asymptomatic

Future 2 consists of having a test, receiving a positive result, and remaining asymptomatic for a period of time. This period between a positive result and disease onset was less imagined (beyond the discussion of reproduction and insurance) and generated rather sparse data. Tom discussed the challenges of explaining to friends how being at risk of HD impacts on him, highlighting the difficulties of communicating to others about a relatively unknown disease.

I just feel like she just didn't really understand it, like ‘Oh well it hasn't affected you and you'll probably be fine and it's in the future’ I just didn't really find those conversations very satisfying and so I stopped. (Tom, Clinic)

Receiving a predictive test result was considered, and planned for, as an important moment. Dee imagined the time immediately after the result and what she would do and feel in the coming days.

It's not going to be pleasant. I'm not under any illusions it's going to be a nice thing to do. It'll take time if I have got it. (Dee, Clinic)

The fact that participants did not often mention this potential future is significant as it is the future they would often be living immediately after they receive their results. Instead, participants concentrated their imagining on a more distant future once they had started to develop symptoms (future 3).

There were also fewer instances in the diary data for this theme. In her diary, Rosie reflected on how a bad news result would impact her decisions about starting a family. Although this was discussed within the clinic consultations also, those discussions often only contained the practical and medical details. In her diary, Rosie contemplates the impact this would have on her relationships and others' expectations.

I may not have a chance [to have a child] – sad that option taken from me and cannot do anything about it. Worried how/if it would affect relationship with partner (albeit he had a child from previous relationship), even concerned about relationship with future in‐laws if can't give them another grandchild. Read stories about different person's experiences of conceiving through IVF and adoption – normally low due to prognosis. Don't know anyone who doesn't have children through choice/medical condition etc. (Rosie, Diary)

Participants mention wanting to make the most of the time before symptoms start. Leah thought about the “healthy time” she would experience and what she wanted to focus on. Her diary entries often contained discussions regarding her contribution to society, where she described “wanting to leave a legacy of achievement and experience” because it then would not be “so shameful to be ill.” She predicted that a bad news result would not affect her plans, but it would enhance her appreciation for the people in her life and encourage her to complete her “bucket list” sooner.

I will be disappointed if I have a high number of repeats and it kicks in at 35–40 because I expected to have more healthy time with my future child and with [partner]. It won't change our plans, but it will make us think of a bucket list for the next 10 years and appreciate each other even more. (Leah, Diary)

In their diaries, participants discussed their motivation to make the most of the time they have left before the symptoms start and contemplated the possible disappointment of hopes not fulfilled.

### 3.3 Imagined future 3: Take a test, receive a positive result, and at the same time, start developing HD symptoms

There was more data imagining a future that explicitly linked a positive predictive test result with the onset of disease compared to futures 2 and 4. It is significant how participants talked about a positive HD test result and the ramifications of that, as imagining a jump from the test result straight to the participant's main concern (developing the clinical symptoms of HD). Therefore, a thread that runs throughout this theme is that of preparing for the worst.

In his clinic consultation, Jacob reflected on how “controversial” it was to be thinking the way he was and so far into the future. Deliberating the decision to find out if he is at risk of HD forced him to bring his distant future significantly nearer.

I'm thinking a lot longer now with mortgages, houses and jobs and stuff like that. I'm paying into a pension scheme at work and actually may not need that in the longer term if there is that. That seems a bit controversial… (Jacob, Clinic)

As with Rosie, participants often used employment as a marker of the significant life changes a positive predictive test result (aligned with symptoms) would bring. Within her clinic consultation, Leah explained how she used the potential future of having a positive HD test result to motivate herself to create the life she really wanted and described the effort that she and her family had made to shape this future:

I had some boring [job], and I was like ‘No, I can do [a different job]!’ so I retrained, started a degree, moved to an area that we wanted to live in. I've already made some structural life changes, with that thinking of ‘is this the life that I wanna continue to lead?’ (Leah, Clinic)

In the consultation, Leah discussed her future as if it was a foregone conclusion. She focused on the practical implications, imagining how the disease would affect her work and how her employer would respond; she was confident of being well supported.

Having the reasonable adjustments and trying to stay in work, would be good, but then I think with the interacting element of my role, I would probably have to stop work sooner, and I don't think they will try and force me to work a role that I wouldn't do. (Leah, Clinic)

Work was also identified as problematic for Dee, who wondered every day how long she had left symptom free and even whether she would live long enough to need her pension: “Do I only have potentially 20 good years left? Do I need a pension?”. As others did, Aaron speculated about the imagined time of onset of his HD symptoms: “It means I wouldn't have 50 years of handiwork, I'd have another ten, or maybe 15 before movements get too bad” (Clinic). Similarly, Rosie hinted at this uncertainty that remained even after getting a positive test result, particularly regarding what symptoms will appear and when. Rosie tried to make sense of this using the templates available to her (as many participants in this study did), comparing her potential future with her father's current situation, where he did not develop symptoms until later in life. But Rosie was also aware of the complexities of HD, and that how HD presents in one family member was not necessarily how it would present in another. With her partner also in the clinic appointment, Rosie explained the significance of being tested before they both commit to marriage. Rosie admitted that it may not be a pleasant thought for them both, but the clinic appointments and the result also functioned to give her partner the opportunity to decide which future was for him.

[Getting married in next couple of years] I can sound horrible and say this. We're making sure you know what you're getting yourself into in terms of me then, really, cos obviously it could potentially be a case where he could be looking [after, taking] care of me in my 30s or 40s. And that's not something generally people would know about. I could be fortunate in terms of being like dad, in terms of not having it until his late 60s, early 70s. (Rosie, Clinic)

The data regarding imagined future 3 from clinic appointments generally contained more practical details about employment and pensions. In contrast, the diary data for future 3 contained evidence of worry and difficult thoughts and feelings. There were more examples in the diary data compared to clinic consultations of participants imagining implications of a positive test result for others. For example, participants frequently mentioned the impact their disease could have on their romantic partners. Jacob wrote in his diary regarding comments read on an HD forum “about the difficulty that people are having looking after their partners.” This led to him worrying about the impact his HD could have on his wife and then having a conversation with her regarding this. In her diary, Dee imagined a time when she would need greater support. She anticipated how HD would affect her and also tried to predict what care she would have to arrange for herself: “Virtually every day the HD situation crosses my mind. What will my life be like? What care will I need to arrange?”

Rosie discussed HD in relation to her partner in her clinic consultations and continued to imagine this in her diary. Importantly, she mentioned something omitted from the clinic appointment about whether her partner would be *able* to care for her if she became ill. Rosie added that this future could also impact children if they decide to have them. Again, Rosie appeared to describe how the risk of HD had forced her to imagine a future she was understandably at odds with.

Will partner be willing/able to care for me? If had children when deteriorate – impact on them emotionally etc and being able to provide for them. Previously had no concern for these precautionary matters. (Rosie, Diary)

Leah wrote an emotive and poignant entry in her diary about an imagined future with HD fueled by comments she found disturbing on a forum. Searching specifically for how men cope with looking after a partner with HD, the results were hard to bear.

I remember obsessively Googling symptoms and reading forums on how husbands were coping with their HD partner. Some of them said really cruel things, about how they [partners] were annoying and unattractive. I literally felt like the floor turned into a black hole swallowing me up, and I felt a sickening sinking sense of dread. I cried and cried and hoped that [husband] wouldn't come downstairs and find me. (Leah, Diary)

This internet research resulted in Leah constructing an alternative future with HD, where she will be cared for by others, not her partner: “I said firmly that I want him to be my husband not my carer, and I'd find a way to pay for a carer to do all of these things for me.” (Diary). Leah used emotive language in her diary entry regarding how society would view her—or maybe how she would view herself—as “disgusting”, not worthy of being loved, and that her HD symptoms would make it hard for others to be around her:

I'm so scared of being ill and useless in society, and I'm scared of getting to that point of being like, ‘what have I done/achieved?’ HD makes me think I'll be mocked and rejected. Why? Because it'll take away my beauty, intelligence & independence – so I'll be nothing. I'll be a pain, a burden, I won't contribute to society. I'll make things awkward. I'd be disgusting and unlovable and would have no place in society. (Leah, Diary)

For other participants, the present was called upon by reflecting on how significant others are managing with the disease. This was the case for Sally who explicitly linked the imagined onset of HD with a sense of feeling trapped. She explained that this is the situation with her father, reflecting on how she would feel if she were at the stage her dad is with his HD, although she also described being grateful that her dad “still has his capacity.” Sally rationalized the possibility of discovering she had the HD gene by saying that, even without it, she could still have health issues as she gets older.

He is frustrated that he cannot get out of bed now. It made me think I would hate to feel trapped in bed. On the other hand, we don't know what old age will bring with other conditions. (Sally, Diary)

In her diary, Rosie described fears about who she may become and also imagined a future where she dies in her 40s.

May not reach old age – suggested cousin may not make 60, and that could be me, or even earlier – worse case starts in 30s and deceased by 40s. Second cousin talking about waiting to finish university – worried about life insurance, starting family – raises my anxieties. (Rosie, Diary)

Aaron imagined no longer being able to use the tools in his shed and how hurt and sad he would be that he could no longer use them. This thought appeared to have tainted how valuable his tools and shed were to him; his treasured tools were turned to junk.

When I do eventually think about it [results appointment], I look at all of my tools and think ‘each one of these will hurt me as I won't be able to hold them properly’. And that makes me feel sad because my shed is my refuge. If it's a bad result, then what's the point in having any of it? It's just junk. (Aaron, Diary)

Two participants wrote about ending their own lives in their diaries. While reading the excerpt from Leah's diary below, we must remind ourselves that Leah had not yet had her test result, however, she had deliberated extensively about her plans once her HD symptoms became more advanced.

Very often my thoughts would come back to the idea of suicide, to reassure myself that I had a way out, and I could keep some dignity. I'll just kill myself when it gets bad. I thought about how I'd do it. I decided morphine. I'd get it and hide it in a place only I knew. (Leah, Diary)

This diary entry from Kayleigh captured her state of mind as she appears angry at her mum for putting her in this position.

Often, I think about it wondering why me? And why did my mum decided [sic] to give birth to me as I would prefer not to be alive dealing with this illness, when I was younger, I had a plan about committing suicide after the illness starts progressing. (Kayleigh, Diary)

As with the clinic data, we see the performative element in the diary data. In her diary, Dee appeared to be irritated by what she perceived the expectations of the clinic appointments to be. She felt a need to perform the role of a capable person who had thought her decision through sufficiently and would be fine regardless of the outcome.

I've been thinking back about my sessions of ‘counselling’ and that is the biggest misnomer ever. The sessions aren't counselling at all, they are patient evaluation sessions. It has basically just been a series of sessions checking me out to see if they think I will lose the plot once I get my results. In many ways it's quite impersonal and I have to defend my decision and prove myself capable of handling the outcome. (Dee, Diary)

It is not perhaps surprising that this imagined future, of being positive for HD and having symptoms, was the most commonly discussed. Although uncertainty was also acknowledged, it is a future that enabled participants to explore their fears about personal decline, loss of independence, and the implications for partners and significant others. However, the fear and worry were less prominent in their discussions with their clinician.

### 3.4 Imagined future 4: Take a test and receive a negative result

A thread that runs throughout this theme is that of hoping for the best. Whereas many participants discussed preparing for a bad news result, hoping for a more immediate, good news result was mentioned less often. Discussion of a good result in the clinic data was most often prompted by the clinician's hope that they would be returning a good news result to the individual at the results appointment, or in reference to discussions regarding informing insurance companies. For example:

If you get tested and you have a good result, then you tell that to the insurer, and they'd put you back to standard average premiums. (Dee's Clinician).

Hope was most often intertwined with preparing for the worst, with participants appearing to mention a good result as a contrast with bad news, not in isolation. Below we see Leah mentioning hope briefly and then situating it within a future with a bad news result.

I'm holding onto some hope [for a negative result] so you know, if it was a bad result, then it would only be human to be disappointed and to be sad about it. (Leah, Clinic)

Participants also described why they were more focused on a bad news result. Because a bad news result was harder to deal with, participants rationalized that a focus on that would be a better way to prepare. It may be that this entwining is because of the serious nature of HD, where “hoping for the best” seems an unlikely outcome.

I'm quite pessimistic normally because then I'm pleasantly surprised when something goes right so I'm leaning more towards having it, but also, I think it's better to be mentally prepared to have it because it's going to be, it's a much bigger thing to deal with. (Aaron, Clinic)

Even though more focus was given to discussing receiving a bad result, clinicians did ask participants “In what way would a good result take adjusting to?” (Dee's Clinician). Dee's reply illustrates the space that deliberating the decision to undergo testing and the weight of potential futures had taken up in her mind and life.

Well just in the sense that it's been part of your life for this amount of time in such a big way, and then it's just like going okay I don't have it, it will just take some adjusting to. (Dee, Clinic)

As with the clinic consultations, there were few mentions of hoping for a good news result in the diaries. Jacob took active steps toward trying to strengthen the hope for a favorable result by searching forums for just that. This imagining was obstructed by finding an entry by a person in a similar position who had received a bad news result, diminishing the hope of a better outcome.

Looked for all good results on the HDA forum and tried to envisage this, stumbled across a bad result from someone in the same situation as me and then decided to stop. (Jacob, Diary)

It was also notable in the diaries how entwined and inseparable were the imaginings of both test outcomes. For example, Aaron appeared to be paralyzed by the equal potential of either outcome and was therefore stuck at the moment with the cognitive burden of a yes/no answer. The 50:50 odds made it impossible to make any distinction between the likelihood of either future becoming a reality.

And in one case, it's a good result and I sit there and imagine how upset I'll be, how relieved I'll be and how celebratory I'll be. But then, in the back of my mind it says, don't get your hopes up because it might not be. And then I go to the other side, where it's a bad result and how upset I'll be and how disappointed and how I'm gonna feel after that. (Aaron, Diary)

One topic that could have sparked hope was that of current and future clinical trials and research regarding the development of HD symptoms. However, most discussions on this topic were instigated by the clinician in clinic consultations. In his diary, Tom discussed current trials in a hopeful manner, for example, “There are treatments being developed for HD which could extend my healthy life expectancy.” However, other examples of research being mentioned in diary data followed a similar pattern whereby they were intertwined with preparing for the worst.

Despite lots of work and advances in HD research I can't help but feel that treatment will potentially be too late for me. (Dee, Diary)

The diary and clinic data both reveal similar insights. Participants explained how they were actively thwarted in their attempts to imagine receiving a negative predictive genetic test result by how intertwined the two futures had become in their mind. Being able to “try out” a future with a good news result is important. As Sally explained, receiving a negative test would be significant and life changing, requiring time to process the result and its implications for the future.

## 4 DISCUSSION

By exploring clinic consultations, diaries, and interviews, we have gained new and important insights into the experiences of participants undergoing predictive genetic testing for HD. We have achieved this through our interest in their lives outside the clinical process alongside their encounters with the world of medicine. This was with the aim of better‐supporting individuals who experience ambivalence and uncertainty within the genetics clinic. Our analysis has identified how the future was imagined by participants and the psychological efforts that went into trying out these different futures, compressing them into the present. The fact that participants attended less to the two futures that were more imminent once a predictive test was taken (positive result before symptoms start and receiving a negative result) is significant, with the majority being more likely to spend time imagining a more distant future with the onset of symptoms. We know that one of the implications of developments in genetic technology has created a separation between a positive predictive genetic test result and the experience of symptoms (Evans et al., 2001). Here, participants are negotiating this as a discrepancy by not imagining this significant period of time after receiving a positive predictive test result. Participants used the imagined futures to navigate the often uncomfortable feelings of uncertainty and ambivalence; we consider the possibility of these insights being drawn upon in the clinical setting.

It is important to understand the process of decision‐making in predictive testing for HD prospectively, as the person tested experiences it, and to highlight the diversity of experiences of different individuals (Cox, 2003; Etchegary, 2006; Ibisler et al., 2017). Purely retrospective studies are limited by participant recall and the reshaping of experiences over time. We identified similar findings to previous prospective studies. For example, participants reporting “having to know” (a self‐evident decision; Cox, 2003), gradual and “evolving” decision‐making (Cox, 2003; Ibisler et al., 2017; Keenan et al., 2015), motivations for testing including the elimination of uncertainty (Ibisler et al., 2017), contemplating suicide (Etchegary, 2006), and threats to self‐identity and changes to relationships with others (Etchegary, 2011). Through diary data, our study adds a layer of insight into how individuals rehearse, as potential scenarios, the different futures that may result from their decision to undergo predictive genetic testing (or not).

Struggling with ambivalence and uncertainty and almost living future 3 (positive test and experiencing symptoms) before it arrives was described by participants as “preparing for the worst.” Even though the proverb “hope for the best but prepare for the worst” was common among participants, little data were generated regarding hoping for the best. This was particularly the case in diary entries where the tone and content appeared more emotionally charged—more pessimistic—compared to clinic discussions. This was true to such an extent that one researcher found analyzing the diary data personally distressing (Ballard, 2020). The diary data revealed topics not discussed in the clinical consultation, such as suicide, the fear of burdening loved ones, and feelings of shame and disgust regarding what it might be like to develop HD symptoms.

How does our data impact considerations about the practice of individuals being encouraged to imagine future 2 (take a test, receive a positive result, and remain asymptomatic) so vividly and the idea of purposefully raising the topic of hope in clinical discussions? Impact bias describes the general tendency we have to overestimate the negative effects on our well‐being (Peters et al., 2014). However, “preparing for the worst” may be a way of coping with distress (Seibaek et al., 2012) as it helps with emotional regulation (Zhu et al., 2023). Here, the individual recognizes hope but spends more time imagining the worst case as preparation for responding more adaptively. Ambivalence may be an adaptive way of coping with uncertainty (Rothman et al., 2017). Here, ambivalence emerges as multiple futures held in the mind simultaneously. This flexibility in thinking may help acknowledge the complexity of the situation, increase acceptance regarding the uncomfortableness, and, in turn, ease the burden on decision‐making.

The current study focused on individuals who had taken steps toward starting the testing process (i.e., had an initial appointment with the clinical genetics team). Individuals who have not yet taken this step may be preserving hope by not finding out their risk status (Quaid et al., 2008), therefore it may be no surprise that hope is less of a focus for individuals who are bracing themselves to find out their genetic status. Participants in this sample might not have allowed themselves to think of this desirable future as even a possibility, or discuss it in any depth because their desire for this outcome was so strong that they could not let themselves express their wish for it. The sadness of inheriting the condition could then be overwhelming. Neglecting the possibility of hope may result in a blind spot regarding the difficult consequences of a “good news” result. Despite receiving such a result, some individuals experience problems in communicating with relatives that they may not have anticipated as well as emotional difficulties, including guilt and a sense of detachment from family members (Godino et al., 2016). Feudtner (2009) encourages the discussion about hope in clinical encounters—especially when complex decisions are being made by individuals—highlighting that this uncovers values that can help guide decision‐making further. Feudtner adds that, by exploring fears as well as hopes, clinicians can help individuals “fortify” the motivation to make and carry out their decisions.

### 4.1 Implications for practice

Our findings provide important material for informing constructive conversations between individuals and the professionals supporting them in the genetics clinic. Our work confirms that how the future is imagined is an important yet underresearched aspect of the decision‐making process. Most prominently, participants spent the least amount of time imagining the more imminent outcomes, either a negative result or a period of good health, when still unaffected, despite a positive result. The personal and familial significance of being tested (or not) for a severe and incurable genetic condition highlights the urgency to acknowledge and explore these hidden futures within the consultation. Our data also show evidence of participants reading disturbing comments on forum websites; therefore, clinicians may want to direct individuals to reliable websites and raise the implications of visiting unmoderated sites.

Our findings raise several questions that require further exploration, including the psychological impact for individuals when they “prepare for the worst,” and the implications if they are encouraged to imagine a future more aligned with “hoping for the best.” In addition, our work has highlighted the potential of diary methods as a viable and useful personal and research tool in the predictive genetic testing process. We would not recommend that individuals be asked to share their personal diaries with the genetic counseling team as that could undermine the benefits. We caution that diaries may be shared with researchers but that those engaged with individuals in the clinic should not expect access if the diaries are to serve as a safe space for personal reflection.

### 4.2 Implications for research

The use of diaries in this research project has been illuminating, offering a qualitatively different insight into individuals' experiences than those gained through the interviews and observations alone. Researchers must be mindful of the time, energy, and commitment keeping a diary can involve and the repercussions of paying particular attention to thoughts and feelings the participant would not normally focus on. This also means that recruitment and retention rates for studies with diary methods can be a challenge. We recommend future researchers use and develop the technique as a method of data generation where appropriate—especially if the method may be preferred by certain underserved groups (see reflections on the diary method below)—and share their experiences with the research community.

### 4.3 Strengths and limitations

A key strength of our study was using diaries as a data collection method. This allowed for an alternative way of facilitating participants' engagement with data collection. This enabled us to capture a degree of insight into the time between the clinic sessions, their “life world.” The vast majority of recommendations in healthcare are made based on data collected in the small periods of time individuals spend in the healthcare environment, the “world of medicine,” whereas most of an individual's journey takes place outside these limited interactions. We also chose to reference themes that were largely absent, strengthening our analysis by highlighting what is not present in the data, as this can lead to novel insights (Ewart & Ames, 2020). Our analysis had limitations, especially as it focused on the psychological aspects of deliberating the decision about testing. In this article, we do not focus on the broader context in which decisions and implications of decisions are being made and experienced. The involvement of others in this process has been reported in a separate paper (Dimond et al., 2022) in which we highlight the intimately linked and complex mechanisms of family and genetics through the lens of “entanglement.” In addition, we acknowledge the limitations of the study's small sample size, meaning we may have not captured the full array of experience, however, the data for each participant were incredibly rich and from three separate sources. Also, our study did not include those who chose not to engage with counseling services or decide not to be tested. Our study did not capture their experiences, despite efforts to encourage those who discontinued the testing process to continue with the research process.

### 4.4 Reflections on the diary method

Using diary elicitation remains an unusual method for understanding participant perspectives. Participants were asked about their reflections regarding the diary method of data collection. Overall, their experience was positive. Completing a diary can be burdensome, time‐consuming, and potentially confronting. While participants acknowledged the personal investment it required, they reported being motivated to complete their diaries, particularly in the context of a research project, by the altruistic element of helping others in the future.

You need space to sit and kind of write so I sort of had to sort of set aside time to do it. (Dee, Interview)

If it is helping me then it's gonna benefit me and hopefully somebody else in the future. (Rosie, Interview)

It was clear that keeping a diary had a therapeutic element for participants, with almost all mentioning this in their interview, although we need to acknowledge there were other research participants who did not complete diaries. The participants in this paper were those who did contribute diaries and they reported that keeping a diary functioned to “get everything off my mind […] out of my system” (Rosie, Interview), “being able to talk about what happened with dad, and that was therapy in itself” (Sally, Interview), and “performing therapy on yourself” (Aaron, Interview). Jacob described how a diary could possibly be useful in reminding individuals why they made the decision they did as he reported not remembering how he had felt even three weeks on from a particular entry.

It's good to reflect I think and put things down and read back. I read back probably about two or three weeks ago, and I can't remember feeling like that, it is good to reflect on it and understand it. (Jacob, Interview)

Two participants specifically talked about how their diary entries were quite candid. Rosie described her diary as being “completely mine,” being able to write exactly what she wanted without writing something “upsetting or offending anybody.” Similarly, Jacob said “If I knew my wife was reading it, I might write it differently,” he added that “it's a personal thing for the research.”

Interestingly, for a multilingual participant, Kayleigh found the diary method helpful because she could think about what she wanted to say and was able to look up translations if needed, something she could not do in a phone call or interview.

When I was writing the diary, I had more time to think or maybe translate it if I did feel something. But when we talk on the phone it's really hard for me to find words, especially when I know I'm doing something important. (Kayleigh, Interview)

We were reassured that the diaries were candid accounts, with participants reporting they were writing details that they would not want their partner to read but were happy to share for research purposes. Significantly, the diary method was preferred to a phone call or interview by one multilingual participant, leading to the conclusion that it may be a method worth exploring to increase accessibility.

## 5 CONCLUSION

These findings highlight the emotional labor that participants undertake between clinic appointments. Even when participants had decided to undergo predictive testing, they were still battling with ambivalence and uncertainty regarding that decision and the psychological discomfort that developed as a result. Through probing clinical consultations, as well as diary data, we have also discovered that expressions of hope are largely absent, and participants ruminate on the less likely outcomes. These findings are useful for clinicians working with this particular at‐risk group to adapt the content and focus of consultations. For researchers, we encourage the use of the diary elicitation method to gain a deeper understanding of individuals' experiences and especially to explore the unseen journey between clinical encounters.

## AUTHOR CONTRIBUTIONS

Authors LMB, SD, RD, AML, and AJC confirm that they had full access to all the data in the study and take responsibility for the integrity of the data and the accuracy of the data analysis. All of the authors gave final approval of this version to be published and agreed to be accountable for all aspects of the work in ensuring that questions related to the accuracy or integrity of any part of the work are appropriately investigated and resolved.

## CONFLICT OF INTEREST STATEMENT

Authors LMB, SD, RD, AML, and AJC declare that they have no conflict of interest.

## ETHICS STATEMENTS

Human studies and informed consent: Approval to conduct this human subject research was obtained by Wales Research Ethics Committee 1. All procedures followed were in accordance with the ethical standards of the responsible committee on human experimentation (institutional and national) and with the Helsinki Declaration of 1975, as revised in 2000. Informed consent was obtained from all individuals for being included in the study.

## Data Availability

The data that support the findings of this study are available from the corresponding author upon reasonable request.
